# Thresholds of Abnormality Perception in Facial Esthetics among Laypersons and Dental Professionals: Frontal Esthetics

**DOI:** 10.1155/2020/8946063

**Published:** 2020-10-17

**Authors:** Raed H. Alrbata, Ayham Kh. Alfaqih, Mohammad R. Almhaidat, Ahmad M. Al-Tarawneh

**Affiliations:** Orthodontic Department, Royal Medical Services, Amman, Jordan

## Abstract

**Aim:**

To find thresholds at which laypersons and dental professionals perceive abnormalities in two facial frontal esthetics variables: facial symmetry and vertical harmony.

**Materials and Methods:**

A baseline frontal image of a young male face with optimum facial proportions was generated from a real image using a photo editing software. Different facial asymmetry images were then generated from this image by manipulating the chin point position at 2 mm increment to the left side. Vertical harmony was perceived through manipulating lower anterior facial height (LAFH) at a ratio of 2% of increased and decreased heights. A total of 120 raters divided equally into four groups of laypersons, general dental practitioners (GDPs), orthodontists, and oral and maxillofacial surgeons (OMFSs) rated these images using an analog scale of 100 mm long. Using ANOVA and Tukey post hoc tests at *P* < 0.05, the image that showed the first statistical difference compared to the baseline was considered as a threshold of abnormality.

**Results:**

The image of 4 mm asymmetry was defined by the laypersons and GDPs as the threshold of abnormality, while the orthodontists and OMFSs realized that at 2 mm. Laypersons and GDPs defined a threshold of 46% as an abnormal decrease in the LAFH and the other 2 groups at 48%. All dental professionals groups rated the image of 54% as an abnormal increase in the LAFH while laypersons perceived that at 56%.

**Conclusion:**

These thresholds regarding frontal esthetics may contribute to the process of establishing proper orthodontic treatment planning that suits the highest facial esthetic standards.

## 1. Introduction

In orthodontics, facial esthetics is a key motivational reason for patients seeking orthodontic treatment [1, 2]. Two main facial aspects are usually considered during examination of orthodontic patients: frontal and profile aspects. Facial symmetry and harmony of vertical thirds are the major variables to consider in the frontal aspect, while the sagittal positioning of the upper and lower jaws are the ones of concern in the profile analysis.

The symmetry of the face represents the balance between the right and left sides. As a certain level of asymmetry might be considered normal, it would be beneficial to know the level at which asymmetry can be perceived as abnormal by different kinds of population, so that intervention is needed. Haraguchi et al. suggested that humans are sensitive to deviations of approximately 4 mm of soft tissue facial asymmetry [3]. Chu et al. found that a minimum of 3 mm was needed for the layperson to notice the face asymmetry in digitally manipulated images [4]. Others found that normal asymmetry range from 0–5.6 mm and the threshold level which laypersons considered acceptable for surgery was around 11.79 mm [5].

Vertical harmony of the face is also crucial as it is considered as one of the main reasons for seeking orthodontic-surgical treatment [6, 7]. In this issue, most of the research found in literature was targeting the profile aspect despite the fact that the usual personal communications are mainly face to face with the main emphasis is on the frontal perspective rather than the profile [8, 9]. Basically, correct anatomic human faces proportions were established at the era of the Renaissance period artists as the distance from hairline to the base of the nose, the base of the nose to the bottom of the nose, and the bottom of the nose to chin should be the same and that the lower third has a proportion of one-third above the mouth to two-thirds below [10]. While some studies found that profiles with increased vertical features were judged to be the most unattractive [11, 12], Cochrane et al. found that long face profiles were ranked as more attractive than Class II and Class III images [13]. Others reported an abnormality threshold of millimeter height for the lower facial third at which laypersons showed that treatment is needed [14].

Orthodontists routinely receive patients from other dental specialties such as general dental practitioners (GDPs) and oral and maxillofacial surgeons (OMFSs) for the purpose of targeting patients' complaints concerning their facial esthetics either by orthodontic treatment alone or as a combination of orthodontic-orthognathic surgery. Accordingly, clarifying the exact thresholds at which these dental professionals and the general public can perceive abnormalities in the main facial aspects is needed. This is to optimize the process of putting together the ideal treatment plan that suits the patient's demands and, at the same time, complies with the esthetic standards.

In this two-part study, the perception of laypersons and dental professionals groups to the main frontal and profile aspects variables will be investigated. The frontal aspect variables, face symmetry and vertical harmony, will be researched in this paper. The aim is to find the thresholds at which these groups could perceive the deviations of these variables beyond the normal limits.

## 2. Materials and Methods

The research protocol was approved by the Royal Medical Services human research ethics committee in November 2018, Amman, Jordan.

A frontal face image for a subject was used in this paper. The subject was selected based on the following criteria: age between 18 and 25 years, no apparent facial asymmetry or vertical face thirds disharmony, and no history of previous plastic surgery. Only one male subject who met the inclusion criteria volunteered to participate and gave his consent by signing a consent form while no females approved due to social reasons.

The image was taken using a DSLR camera (Nikon D3200, Lens 85 mm, and ring flash). It was modified to get the optimum frontal facial proportions (transversely, symmetrical left and right halves of the face and vertically, equal thirds of the face height) to be used as a baseline for the manipulations needed for this study at 12.5% reduction compared to the original life-size image using the software Adobe Photoshop (CS6 version 13, Adobe Systems Inc., CA, USA).

### 2.1. Manipulation of the Baseline Image to Affect Facial Symmetry (Figure 1)

Five frontal images were created by modifying the deviation of the mandible and chin of the baseline image to the left side by incremental measures of 2 mm from 0 mm (Baseline) to 10 mm relative to facial midline so that facial symmetry was compromised. The facial midline, based on the work of Arnett and McLaughlin, was drawn using a line through the philtrum of the upper lip and the center of the nasal bridge which is assessed as half the distance between the inner canthi of the eyes [15]. Only the chin point position, which should normally be located at the line, and ramal length with accompanied lips canting were precisely manipulated by moving them digitally in a left-upward direction with the same incremental measure relative to the facial midline without affecting other facial standards.

### 2.2. Manipulation of the Baseline Image to Affect Vertical Harmony of the Face (Figure 2)

The area of concern in this section was the lower anterior facial height (LAFH) which represents the area from the bottom of the nose to the chin point. Two variations were researched here. As the clinical LAFH represents half that of the facial height (combining the LAFH and middle facial height), manipulation of the baseline frontal image was performed to produce 4 images of increased LAFH of 2% intervals (52, 54, 56, and 58%) and 4 images of decreased LAFH of the same sequence values (48, 46, 44, and 42%) relative to the face height. The procedure of image manipulation using the computer software commenced by digitally defining multiple points comprising the lower face part at the baseline image to freely move and make changes to this part without affecting the other face parts. Considering the face height, the defined part was either vertically shortened or elongated for the purpose of respectively reducing or increasing the lower face height at the 2% interval. Undoubtedly, the average ratio between upper and lower lips lengths was maintained for all images.

The incremental manipulations in all variables were set as minimal as possible for the purpose of precisely defining the thresholds at which the first abnormality degree could be perceived by the different raters while at the same time maintaining more realistic soft tissue facial changes. For each of the 3 variables, the manipulated images along with the baseline were given different symbols of 2 different letters and randomly arranged at a separate paper (A3) in landscape orientation for evaluation at the same session to allow fair comparisons to be made as was performed by previous studies [16, 17]. A duplicate of one of the images was used to assess intrarater reliability in each variable.

### 2.3. Participants

A total of 120 raters participated in the study (Table 1). The selection criteria for the laypersons were as follows: having a Bachelor degree in any field except medicine or dentistry and no history of orthodontic or orthognathic surgery treatment. The GDPs: has no higher education in any dental field. For orthodontists and OMFSs: have experience of at least 3 years in their fields. An equal number of males and females were considered for all participants except for the OMFSs who were almost males as in Jordan, the females OMFSs are few in numbers. All the raters were from the same country and of Arabic origin.

The sample size was determined using a pilot study. The effect size was estimated at 0.95. On the basis of a significance level of alpha 0.05, the sample size was calculated to achieve 80% power and showed that 30 subjects for each group were necessary.

The determination of the subjective esthetic values in each variable was accomplished by using a visual analog scale of real 100 mm long and the raters used their own esthetic values to rank each image from “0: least attractive” to “100: most attractive” by pointing out their values at the scale using a marker. Each rater was given a booklet to fill in his own esthetic perception values based on the fact that the more pleasant and attractive face images should be given high-rank numbers compared with the less pleasant and attractive ones. The booklets were having the 3 pages with the manipulated images of the 3 variables (Asymmetry, increased LAFH and decreased LAFH), a letter of appreciation for participation in the study, and the page of the rating scales with the symbols printed for each image at the end of each scale. The rating result for an image was obtained by measuring the distance in millimeters between the least attractive zero point to the rank point made by the rater using a ruler. Only one orthodontist performed this procedure to avoid interexaminers errors.

To check for reliability of the analog scale measurements by the orthodontist, he was asked 2 weeks later to remeasure the esthetic values for 10 raters from each group for the asymmetry variable. The results found were acceptable overall with inter- and intraclass correlation coefficients of, respectively, 0.87 and 0.89 minimum. All values were transferred to an excel file for the purpose of the needed analyses.

### 2.4. Statistical Analysis

All statistical tests were performed using the statistical software SPSS v21 (IBM Corp., Armonk, NY, USA) at the *P* < 0.05 level of significance. ANOVA and Tukey post hoc tests were used to assess differences in the rating of the manipulated images compared to the baseline in each variable. Intra- and interclass correlation coefficients were used to determine the agreement between the duplicate images used in the study and for the reliability test concerning the analog scale measurements.

## 3. Results

Mean values and standard deviations (SDs) for the esthetic values of the asymmetry variable are shown in Table 2. All raters' groups gave the highest esthetic values for the baseline image (0 mm). For each group, an inverse relation was clearly shown between the esthetic values registered by the raters and the values of incremental manipulations performed for the images. As the mean values started to decrease along with the increase in the incremental mm manipulations, using the Tukey post hoc tests, the first statistical significance appeared was registered as the threshold of abnormality perceived by the specific group. Orthodontists and OMFSs were very precise in significantly detecting the abnormality in facial symmetry at 2 mm chin deviation while the other 2 groups (laypersons and GDPs) perceived that at 4 mm (*P*=0.000).

Table 3 shows the mean esthetic values for the decreased and increased LAFH variables. All groups gave higher esthetic values for the baseline image (50%) in the 2 variables except the orthodontists group who showed a higher esthetic mean for the 52% image. However, this was not statistically significant as compared with the baseline (*P*=0.735). The first statistical significance appeared and registered as the threshold of abnormality perceived in the decreased LAFH images was 46% for the laypersons and GDPs groups and 48% for the orthodontists (*P*=0.004) and OMFSs (*P*=0.000), while in the increased LAFH images, the thresholds for the 3 professional groups (GDPs, Orthodontists, and OMFSs) were the same (54%) being more precise as compared with the laypersons who showed that at 56%.

High levels of reliability were found between the duplicate images for the three variables analyzed and between the two baseline images which were separately rated for the decreased and increased LAFH variables as all intraclass correlation coefficients were greater than 0.85.

## 4. Discussion

The subjectivity of human face beauty is an important issue. What is judged as pleasant by a person might not be that by another one. This is also the case between dental professionals. For this, orthodontists need to keep updated with how laypersons and dental professionals, who take part in the process of establishing the orthodontic treatment plan for a patient-perceive any degree of deviation beyond normality in the face beauty.

A colored real image of the face rather than using drawings or silhouettes was used for all variables investigated. Although this kind of images might distract the raters due to the presence of other face components, such protocol was found to add more realism to the representation of the facial esthetics and that these components were controlled in this study by maintaining the same features for all images manipulated [18].

The most prevalent type of asymmetry in the orthodontic clinic was found as the one that affects the ramus and menton parts of the mandible, as classified by Hwang et al. [19]. In our study, the incremental manipulations of the symmetry variable were targeting this type without affecting other parts of the face. As shown in Table 2, and in agreement with other researches [20, 21], the baseline image gave the highest esthetic means compared to the manipulated images at which, after that, the means were declining along with the increase in the incremental manipulations for each rater group. Orthodontists and OMFSs were more sensitive to perceive abnormality in the face symmetry at 2 mm chin point deviation than the GDPs and laypersons who considered this as normal up to 4 mm. For the dental professionals, this may be due to the fact that orthodontists and OMFSs usually encounter and treat more facial asymmetry cases than the GDPs so that they were more precise in detecting the abnormality.

The results of this study may coincide or disagree with other researches which reported thresholds of 3 mm asymmetry for laypersons as found by Chu et al. [4] and an average of 5.6, 3.6, and 4.4 mm for laypersons, orthodontists, and GDPs, respectively, as found by others [5]. However, this might be affected by several factors such as ethnicity [22, 23] and the value of incremental manipulations adopted, as it is the case in the study of Naini et al. [24], who found that deviations of less than 5 mm were not considered important but this was due to the 5 mm incremental manipulation which is larger than the more precise increment of 2 mm we have used. Also, Haraguchi et al. found that the orthodontists' threshold of asymmetry perception was approximately 4 mm [3]. It might be the impact of ethnicity as the baseline images used were of Japanese sample.

For the vertical harmony of the face, little research was found to define thresholds of abnormality. Varlik et al. reported a millimeter height range of the LAFH at which beyond this range, the esthetic perception was affected [14]. However, considering ratios as it is the case in our study rather than the millimeter height might be more sensible to consider so that more comprehensive face perception could be performed.

All raters groups ranked the baseline image at the top of the images in both LAFH variables except that orthodontists gave the 52% image a higher esthetic value but that was not statistically significant. These results are in harmony with observations by Johnston et al. [12] and Erbay and Caniklioğlu [25]. Clinically, these values may establish a base at which orthodontic-orthognathic treatment planning could be implemented. For example, slightly increasing LAFH (52%) for a patient would be very pleasant by orthodontists but not that optimum for OMFSs during planning vertical positioning of both jaws in orthognathic surgery. However, based on the results above, an orthodontist will not be disagreed if the 50% ratio is demanded by the OMFS or the patient.

Orthodontists and OMFSs were more critical in detecting the reductions in the LAFH compared to the other two groups. In the increased LAFH images, all dental professionals perceived the abnormality at 54% level, being more sensitive than the laypersons. Although it seems normal to find that laypersons were more superficial in detecting abnormalities in the LAFH changes compared to the dental professionals, others showed contradictory results of that both orthodontists and laypersons were equally sensitive to the LAFH changes [26].

It should be noticed that many of the images were rated affirmatively and given values of more than 50 mm of a 100 mm scale which means that they are still perceived positively by the different raters. Our target was to find the image at which the first statistical significance compared with the baseline image could be seen without giving importance to the ranking value in itself. Overall, while such numerical esthetic thresholds are not that much different between groups and may not influence in particular the patients' decisions concerning their orthodontic treatment objectives; however, there are patients who are strict in their choices and ask for beauty perfection. Hopefully, these thresholds will represent the reference at which orthodontic treatment could be set up properly with the agreement of both the orthodontist and patient.

A clear word said at the end is that the incorporation of a female subject in this study was a challenging desire to implement. As only one young male 2D downsized image was used as a baseline, whether selecting a female or another male with different age or a 3D image of actual sized will affect the results might need to be investigated. The impact of ethnicity on the thresholds resulted in a crucial issue and should not be overlooked when considering such results. Also, differences in male to female raters' perception were not tested. Although this is important and despite that, an almost equal male to female ratio was chosen, this may be to avoid expanding the objectives researched in the study and hopefully, this issue will be investigated in the near future.

## 5. Conclusions

Frontal esthetics of the face were researched in terms of the thresholds at which laypersons and dental professionals could perceive abnormality in 2 main variables, facial symmetry and vertical harmony of the face. It was found that

- Orthodontists and OMFSs were more sensitive to perceive abnormality in the face symmetry at 2 mm chin point deviation than the GDPs and laypersons who defined that at 4 mm.
- Also, orthodontists and OMFSs were more critical in detecting the reductions in the LAFH at 48% compared to the other two groups with a threshold value of 46%.
- In the increased LAFH images, all dental professionals groups perceived the abnormality at the 54% level, being more sensitive than the laypersons with a threshold of 56%.

## Figures and Tables

**Figure 1:**
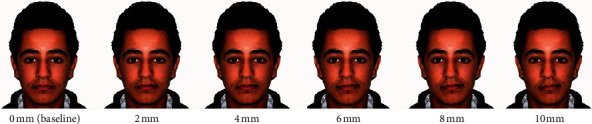
Baseline and manipulated images to affect the symmetry of the face.

**Figure 2:**
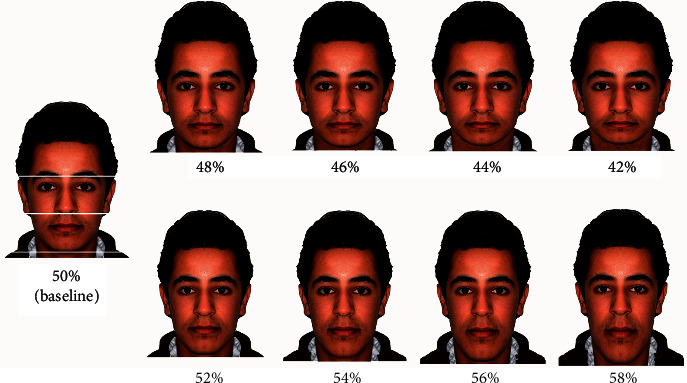
Baseline and manipulated images to affect decreased and increased LAFH ratios to the face height.

**Table 1:** Rater demographics.

Rater group	Number	Mean age **(**yr**)**	Age range **(**yr**)**	95% CI^*∗*^	Males *N* (%)	Females *N* (%)
Laypersons	30	31	21–41	29–33	15 (50)	15 (50)
GDPs	30	36	25–47	34–38	15 (50)	15 (50)
Orthodontists	30	38	27–49	35–41	15 (50)	15 (50)
OMFSs	30	39	30–48	37–41	28 (93)	2 (7)

^*∗*^Confidence Interval, N: Number.

**Table 2:** Means and SDs of the esthetic values for the baseline face symmetry image and the manipulated images as perceived by the four raters groups.

Images	Laypersons (*N* = 30)			GDPs (*N* = 30)			Orthodontists (*N* = 30)			OMFSs (*N* = 30)		
	Mean	SD	*Sig*	Mean	SD	*Sig*	Mean	SD	*Sig*	Mean	SD	*Sig*
0 mm (baseline)	75.41	15.76	—	89.06	7.91	—	88.32	6.12	—	89.30	9.09	—
2 mm	67.68	15.95	866	80.39	11.98	0.627	76.36	8.41	0.008^*∗∗*^	72.80	18.05	0.007^*∗∗*^
4 mm	51.05	24.24	0.000^*∗∗*^	62.33	18.75	0.000^*∗∗*^	59.54	18.06	0.000^*∗*^	60.00	13.31	0.000^*∗*^
6 mm	50.05	20.50	0.000^*∗*^	36.22	20.70	0.000^*∗*^	48.04	17.06	0.000^*∗*^	46.50	10.43	0.000^*∗*^
8 mm	40.36	22.88	0.000^*∗*^	25.44	13.24	0.000^*∗*^	34.64	14.02	0.000^*∗*^	41.85	15.05	0.000^*∗*^
10 mm	30.86	16.97	0.000^*∗*^	18.11	12.12	0.000^*∗*^	28.96	13.11	0.000^*∗*^	38.45	13.23	0.000^*∗*^

^*∗*^indicates significance at *P* < 0.05 as a result of Tukey post hoc test. ^*∗∗*^indicates first significance appeared compared to the baseline image.

**Table 3:** Means and SDs of the esthetic values for the baseline LAFH image and the manipulated decreased and increased LAFH images as perceived by the four raters groups.

Images (%)	Laypersons (*N* = 30)			GDPs (*N* = 30)			Orthodontists (*N* = 30)			OMFSs (*N* = 30)		
	Mean	SD	*Sig*	Mean	SD	*Sig*	Mean	SD	*Sig*	Mean	SD	*Sig*
42	35.95	28.43	0.000^*∗*^	18.83	18.73	0.000^*∗*^	29.32	13.06	0.000^*∗*^	28.65	17.36	0.000^*∗*^
44	41.36	24.01	0.001^*∗*^	26.17	18.29	0.000^*∗*^	37.54	13.79	0.000^*∗*^	39.50	17.04	0.000^*∗*^
46	46.86	24.67	0.010^*∗∗*^	36.56	27.93	0.000^*∗∗*^	43.25	16.62	0.000^*∗*^	49.15	16.90	0.000^*∗*^
48	55.41	25.38	0.218	67.67	20.81	0.435	65.75	12.55	0.004^*∗∗*^	61.90	19.28	0.000^*∗∗*^
50 (baseline)	71.75	15.25	—	80.58	11.20	—	78.68	10.40	—	84.70	12.07	—
52	70.36	18.88	1.00	79.50	14.37	1.00	83.32	10.03	0.735	76.85	11.29	0.646
54	58.86	26.82	0.498	47.11	21.19	0.000^*∗∗*^	53.57	21.27	0.000^*∗∗*^	54.45	21.51	0.000^*∗∗*^
56	37.45	27.60	0.000^*∗∗*^	18.67	13.22	0.000^*∗*^	20.93	7.24	0.000^*∗*^	35.85	18.96	0.000^*∗*^
58	23.05	24.05	0.000^*∗*^	8.83	8.05	0.000^*∗*^	13.21	9.71	0.000^*∗*^	14.95	11.50	0.000^*∗*^

^*∗*^indicates significance at *P* < 0.05 as a result of the Tukey post hoc test. ^*∗∗*^indicates the first significance appeared compared to the baseline image.

## Data Availability

The SPSS data file used to support the findings of this study are available from the corresponding author upon request.
